# The effect of GV-58, a calcium channel modifier, on synaptic transmission at the larval *Drosophila* and crayfish neuromuscular junctions

**DOI:** 10.17912/micropub.biology.001402

**Published:** 2024-12-23

**Authors:** Jackson Schwamburger, Kaitlyn Brock, Robin Cooper

**Affiliations:** 1 Biology, University of Kentucky, Lexington, Kentucky, United States

## Abstract

GV-58 is known to increase the opening time of the mammalian P-type calcium channel in presynaptic motor nerve terminals. GV-58 is suggested as a therapeutic agent for dampening the symptoms of amyotrophic lateral sclerosis. To further understand the mechanisms of GV-58 actions, the *Drosophila* and crayfish neuromuscular junctions were used as models. Their presynaptic calcium channels are a P-type based on pharmacology profiles. However, exposure of GV-58 (1mM) did not produce any consistent alteration in synaptic transmission in these two preparations. It is possible that the molecular structure of the P-type channels is different in the *Drosophila* and crayfish.

**Figure 1. The effect of GV-58 on synaptic transmission at the Drosophila and crayfish neuromuscular junctions.**
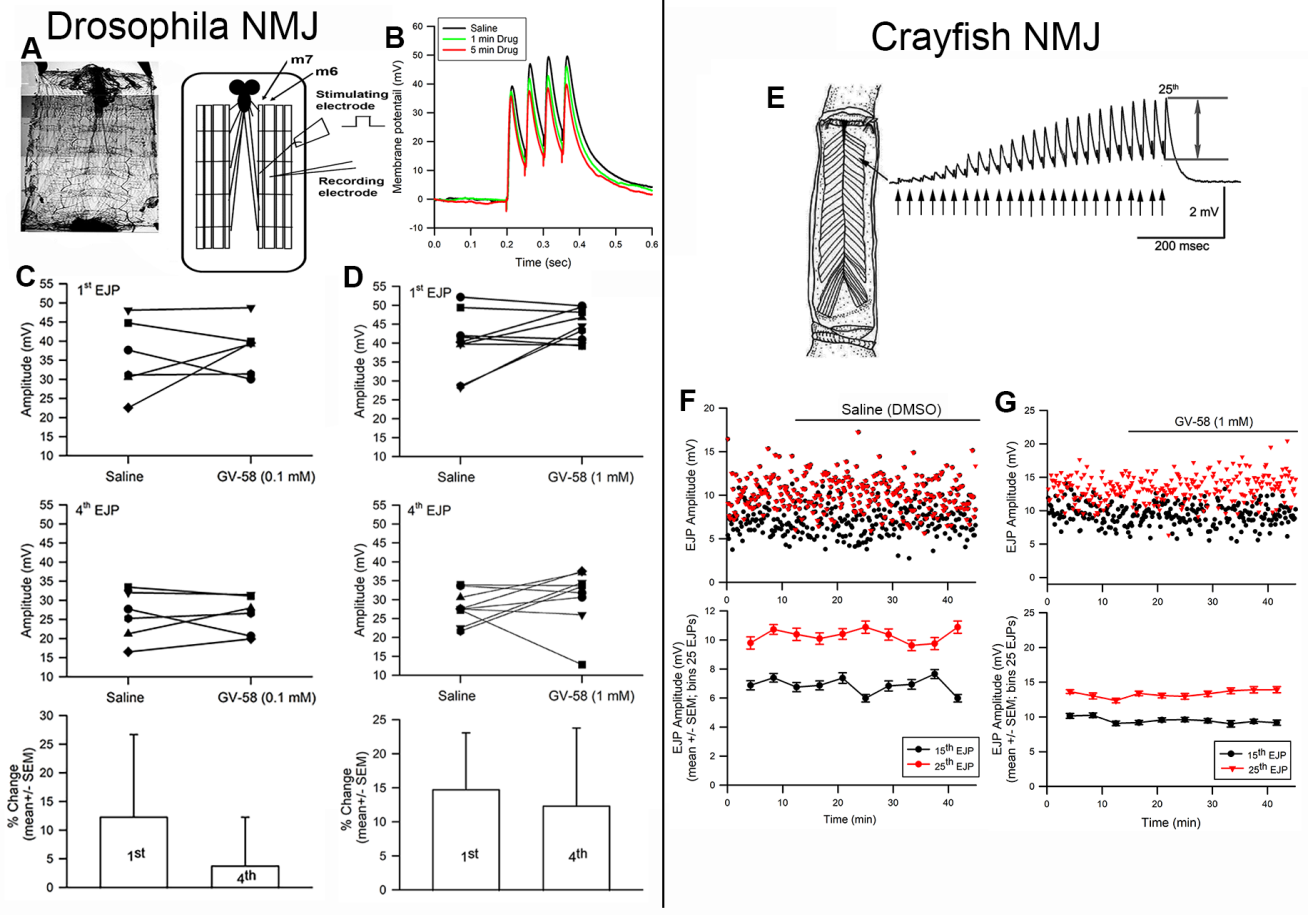
(A) Dissection and recording of synaptic excitatory junction potentials (EJPs). (B) Four stimulation pulses every 10 seconds, the 1 ^st^ and 4 ^th^ EJP amplitudes were measured. (C) The amplitudes of the EJP saline and exposure to 0.1mM of GV-58 or to (D) 1 mM of GV-58. (E) The opener NMJ of the walking leg of a crayfish when stimulated at 40 Hz for 25 stimuli. (F) The 15 ^th^ and 25 ^th^ EJP amplitudes of a representative preparation in saline and (G) during exposure GV-58 (1mM).

## Description

GV-58 is a compound that is suggested to target presynaptic voltage gated Ca ^2+^ channels (VGCCs) of the “P-subtype”, or also known as Cav2.1. The VGCCs at the presynaptic motor nerve terminals of the larval *Drosophila* and crayfish neuromuscular junctions (NMJs) are both of a “P-subtype” (Araque et al., 1994; Kawasaki et al., 2004) . GV-58 has been suggested to be used as a possible therapeutic agent to treat symptoms of amyotrophic lateral sclerosis (ALS). In the early onset of ALS synaptic efficacy is dampened. This is a critical time to help promote synaptic strength. Still there are no significant therapeutic treatments to retard the progression and prevention of motor neuron death. Selective enhancement of synaptic transmission at NMJs would aid in alleviating these early symptoms. Reports suggest that GV-58 is a modifier of the P-type calcium channels and prolongs the opening time of the channel when it opens due to voltage. Thus, more Ca ^2+^ would enter the presynaptic nerve terminal and result in enhanced amount of transmitter release when the nerve is stimulated.

This investigation examined the effects of GV-58 on synaptic transmission at the model *Drosophila* and crayfish NMJs. Since synaptic transmission is graded, subtle changes in the presynaptic Ca ^2+^ influx are readily measured by the amplitude and short-term facilitation (STF) of the excitatory postsynaptic potentials (EJPs) (Katz and Miledi, 1968;). STF is a transient increase in the amplitude of subsequent EJPs when more than one stimulus is rapidly given so that the residual Ca ^2+^ will increasewith each stimulus within the presynaptic terminal (Cooper and Cooper, 2009) .

Exposure of GV-58 to the NMJs of the larval *Drosophila* body wall muscle and crayfish opener muscle did not reveal any consistent trend in altering synaptic transmission. The synaptic responses were mixed at the larval Drosophila NMJ, with some increasing the amplitude of the EJPs and some decreasing for both 0.1 mM and 1.0 mM exposure. However, for the crayfish NMJs there were no significant trends in the amplitudes of the EJPs within the trains for stimuli during the STF responses before or during exposure to GV-58. Since small changes in the extracellular Ca ^2+^ concentration in the bathing saline as well a buildup in the internal concentrations during STF are known to produce significant changes in the amplitudes of the EJPs in both preparations, it is concluded that GV-58 does not likely enhance the concentration of Ca ^2+^ in the presynaptic terminals significantly during evoked stimulation to impact synaptic transmission.

GV-58 has been shown to have an enhancing effect in synaptic transmission at mouse NMJs (Li et al., 2023; Ojala et al., 2023) , but it has also been shown to activate voltage gated Na+ channels (Cho et al., 2022) . Though the pharmacological profile indicates that the presynaptic calcium channels in the crayfish and *Drosophila* are deemed a P-type, it might not be relatable to the same “P-type” as classified in mammalian preparations. A known blocker of P-type voltage-dependent Ca ^2+^ channels is the venom of the funnel-web spider *Agelenopsis aperta* which contains the peptide omega-Aga-IVA. This is why these channels were classified as such in the crayfish and other preparations (Araque et al., 1994) . Also, physiological measures in the current voltage relationships of the crayfish motor neurons indicted a relationship between P-type calcium channels of mammals (Hong and Lnenicka, 1997; Tsien et al., 1988) . The molecular identification of all the voltage gated ion channels within the presynaptic motor nerve terminals for the crayfish and *Drosophila* have yet to be fully identified and compared. Additionally, accessory proteins associated with the channels, which can be influenced by pharmacological agents, may also have a role in the function of the channels. Given the P-type Ca ^2+^ channels of mammals are of the pharmacological nomenclature it would be of interest to obtain protein sequence comparisons among crayfish, *Drosophila* and mammalian models, along with accessory proteins, so further comparisons can be made as for potential screening of modulators for these channels to better understand the molecular mechanisms of action.

## Methods

The experimental paradigm was to measure the effects before and during exposure to GV-58 on the amplitude and facilitation of the synaptic responses at the larval *Drosophila* and crayfish NMJs. GV-58 was dissolved in 0.1 % DMSO prior to adding to the saline. The saline control also contained 0.1 % DMSO.

***Larval Drosophila melanogaster***

Early 3 ^rd^ instar Canton-S *Drosophila melanogaster* larvae were used for electrophysiological measures of muscle m6. Canton-S strain was obtained from Bloomington Drosophila Stock Center (stock # 64349). Transmembrane potentials were obtained with sharp intracellular electrodes (30 to 40 megaOhm resistance) filled with 3 M KCl. Fly saline haemolymph-like 3 (HL3) was used: (in mmol/L) 70 NaCl, 5 KCl, 20 MgCl _2_ , 10 NaHCO _3_ , 1 CaCl _2_ , 5 trehalose, 115 sucrose, 25 N,N-bis(2-hydroxyethyl)-2-aminoethane sulfonic acid (BES) and pH at 7.1. An Axonclamp 2B (Molecular Devices, Sunnyvale, CA, USA) amplifier and 1 X LU head stage were used. The EJPs and spontaneous mEJPs were collected and analyzed with LabChart 7.0 (ADInstruments, USA).

***Crayfish Opener muscle***

Experiments were also performed using *Procambarus clarkii* bought directly from Kyle LeBlanc Crawfish Farms, Raceland, LA USA, 70394. Crayfish measuring 6–10 cm in body length and 12.5–25 g in body weight were used. Details of the dissection and electrophysiological recordings of the opener neuromuscular junction of the walking legs were previously described in video format (Cooper and Cooper, 2009) . The crayfish saline used is in mM: 205 NaCl, 5.3 KCl, 13.5 CaCl _2_ ·2H _2_ O, 2.45 MgCl _2_ ·6H _2_ O, and 5 HEPES adjusted to pH 7.4. The excitatory axon innervating the opener muscle in the crayfish was stimulated in the meropodite by placing a branch of the leg nerve into a suction electrode connected to a Grass stimulator. Stimulation was with 40-Hz trains of 25 pulses in duration. Intracellular recordings were obtained in the same manner as described for the *Drosophila* preparation above. The nerve was stimulated at 40 Hz to facilitate the amplitudes of the EJPs to reliably measure them from baseline noise.
